# Pains in Chest and Back

**Published:** 1883-04

**Authors:** 


### PAINS IN CHEST AND BACK.

AMBRA: Lancination in chest extending to the back.

*Oppression* of chest extending to the back, between the scapulæ; relieved a short while by eating.

BRYONIA: During an inspiration, stitch through to the scapulæ.

CALCAREA: Violent stitches from the thoracic cavity, extending through the spinal column and coming out between the scapulæ.

CAMPHORA: Painful drawing stitches through and between the scapulæ, extending into the chest, when moving the arms, for two days.

CAUSTICUM: Stitches commencing deep in chest and coming out at the back.

COLCHICUM: Lancinating tearing, deep in right breast, through to back.

DULCAMARA: Dull stitching pain in right side of chest, in region of third rib, especially when pressing the part, when the pain went to small of back and extended between the shoulders.

Lancinating pain from middle of sternum to dorsal spine, when sitting; it goes off when rising.

INDIGO: Severe, sharp stitch in the middle of the sternum, passing through the chest, when sitting.

KALI BICHR.: Stitches under sternum through to back.

Pain extending from small of back to nape of neck and shooting through to sternum, preventing him from working for four weeks. Stabbing from third cervical to fifth dorsal vertebra, striking forward through chest to sternum, increased on motion, with inability to straighten the spine after stooping.

LAUROCERASUS: Stitches in chest from back to sternum.

Stitches in sternum, also in middle or in lower part; also extending to back, in evening, during an inspiration.

MERCURIUS: Aching pain inside of sternum, extending through to back, and being even felt during rest, but worse when walking in evening; afterward the place felt painful as if bruised.

When *sneezing and coughing*, between the acts of respiration, he feels a stitch in the anterior and superior portions of the chest, extending through to back; chest feels contracted and squeezed together by the stitch.

NITRIC ACID: Violent stitch in upper part of and within the right ribs, through abdomen and back.

PÆONIA: Throbbing through right chest and extending posteriorly up to nape of neck, where the throbbing terminates in intermittent pinching.

PHELLANDRIUM: Violent stitch through right mamma near sternum, through to back between shoulders, and then striking downward into right side of os sacrum, which is very painful on drawing breath after dinner.

PHOSPHORUS: Cutting from middle of sternum to right scapula, worse during an inspiration, *less* during motion.

RAPHANUS: Pain in chest, particularly when eating and coughing, less when drinking, the pain being of an aching and sticking character, extending from pit of stomach to throat-pit and frequently to back.

RHUS RAD.: Drawing and stitching pain, extending from left side of chest, near nipple, to left scapula, aggravated by coughing, sneezing, yawning, etc.

SILICEA: Stitches in chest and sides (right) through to back.

SPIGELIA: Lancinating pain below left nipple and extending into region of scapula and upper arm, more violent during deep inspiration.

SULPHUR: Stitch from right chest to scapula.

ZINCUM: Stitch in upper part of sternum, extending to left lumbar region, with dread of stooping, early in morning.

Stitches under left scapula, extending to forepart of left region of chest.

### HERING'S GUIDING SYMPTOMS.

The fourth volume of this work is said to be in press, and the editors hope to issue two volumes a year until completed.

### HERING MEMORIAL VOLUME.

The gentleman in charge of the Hering Memorial Volume announce that it is nearing completion. The biographical portion, by Wm. Furness, D. D., has gone to press.

